# Comparative analysis of hemolysis incidence across 3 different pulsed field ablation systems

**DOI:** 10.1016/j.hroo.2026.04.009

**Published:** 2026-04-16

**Authors:** Hiroyuki Miyazawa, Satoshi Yanagisawa, Yasuya Inden, Taishi Fukushima, Takehiro Hiramatsu, Kentaro Adachi, Tsubasa Teraoka, Ryusuke Ota, Kiichi Miyamae, Yuki Tanaka, Masafumi Shimojo, Yukiomi Tsuji, Toyoaki Murohara

**Affiliations:** 1 Department of Cardiology, Nagoya University Graduate School of Medicine, Nagoya, Japan; 2 Department of Advanced Cardiovascular Therapeutics, Nagoya University Graduate School of Medicine, Nagoya, Japan

**Keywords:** Atrial fibrillation, Pulsed field ablation, Hemolysis, Myocardial injury, Acute kidney injury

## Abstract

**Background:**

Pulsed field ablation (PFA) is associated with a risk of hemolysis; however, the differences among 3 single-shot PFA systems remain unclear.

**Objective:**

This study aimed to examine the differences in hemolysis among 3 PFA systems.

**Methods:**

We assessed changes in hemolytic, myocardial, and renal biomarkers at baseline and 24 hours after ablation in 131 patients with atrial fibrillation treated with 3 PFA systems (VARIPULSE, n = 26; PulseSelect, n = 62; FARAPULSE, n = 43), as well as in 23 patients undergoing radiofrequency ablation (control) and 8 patients without ablation (nonablation control).

**Results:**

Baseline characteristics were comparable across the groups. Haptoglobin levels decreased from 97.3 ± 52.1 to 49.9 ± 40.1 in the VARIPULSE group, 96.7 ± 41.9 to 53.5 ± 31.2 in the PulseSelect group, and 90.6 ± 43.8 to 27.3 ± 18.6 in the FARAPULSE group (all *P* < .001). Compared with the control group, both hemolysis and myocardial injury were more pronounced in the PFA groups, with the greatest severity observed in the FARAPULSE group. No significant deterioration in renal function was observed in any group after ablation; however, 2 cases of stage 1 acute kidney injury occurred in the FARAPULSE group, both of which fully resolved with hydration. Postablation haptoglobin levels exhibited a significant negative correlation with the number of applications across all PFA groups. Multivariate regression analysis identified the application ratio (total number of applications/manufacturer’s recommended applications) and body mass index as independent predictors of reduced haptoglobin levels.

**Conclusion:**

Intravascular hemolysis is a pertinent concern after PFA and is associated with the number of applications and body mass index, with potential differences across the 3 PFA systems.

## Graphical abstract

**Figure:**
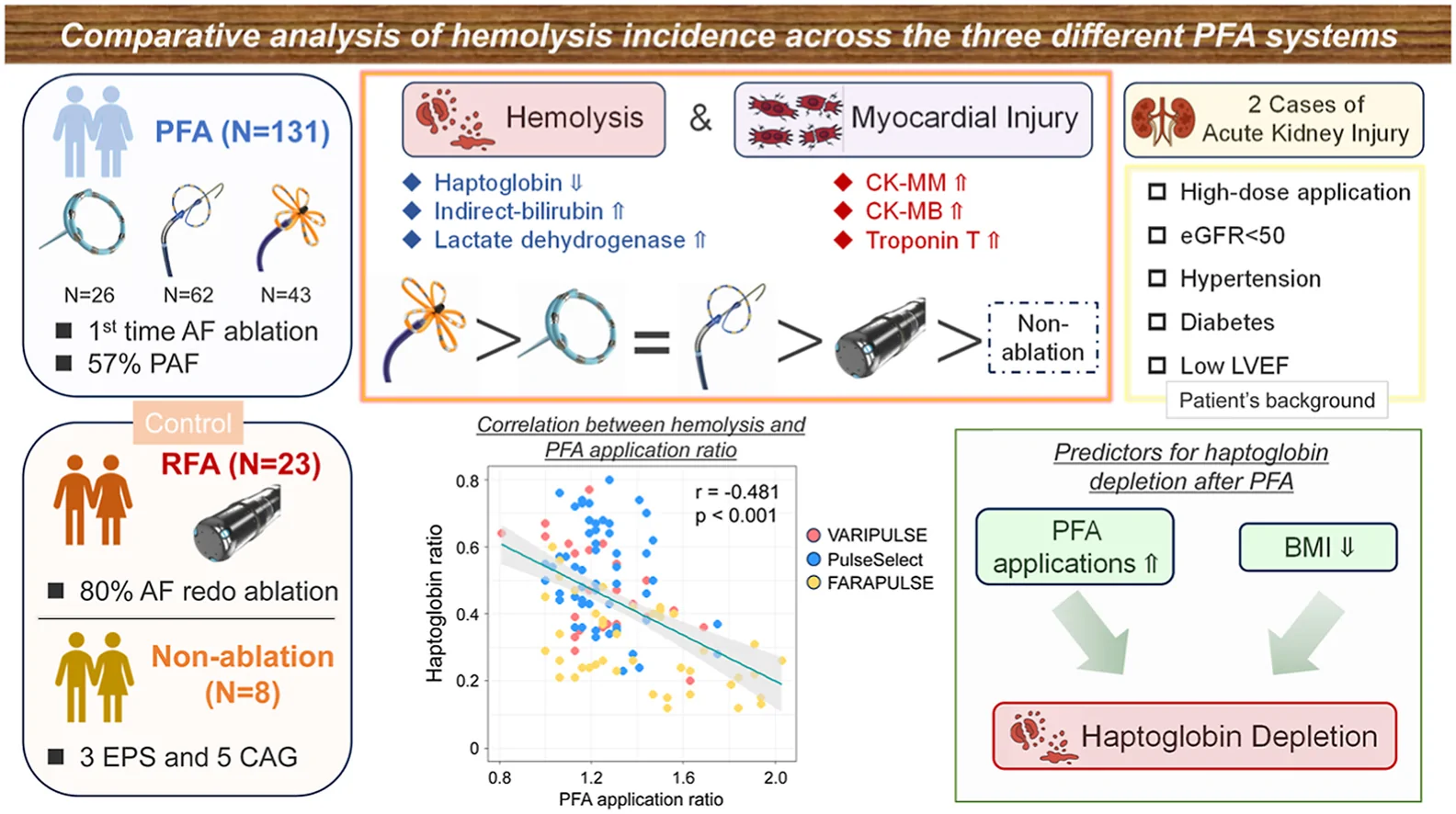

**Key Findings**

▪ Intravascular hemolysis occurs frequently after pulsed-field ablation (PFA), with greater severity than radiofrequency ablation and no changes observed in nonablation procedures.
▪ Among 3 commercially available single-shot PFA systems, FARAPULSE was associated with the greatest degree of hemolysis and myocardial injury, whereas PulseSelect showed the least changes.
▪ The degree of hemolysis was significantly associated with procedural exposure, with a clear correlation between the number of PFA applications and haptoglobin depletion across all systems.
▪ The application ratio and body mass index were independent predictors of hemolysis, suggesting both procedural and patient-related determinants.
▪ Although hemolysis was common, clinically significant renal dysfunction was rare, indicating that careful procedural optimization—particularly minimizing unnecessary applications—may mitigate risk.

## Introduction

Pulsed field ablation (PFA) has been reported to possess a superior safety profile compared with thermal energy ablation, attributed to its tissue selectivity and nonthermal energy characteristics.^1^ Nevertheless, hemolysis and consequent acute kidney injury (AKI) have emerged as unique complications associated with PFA.^1^^,^^2^ Recent studies have reported hemolysis and occasional AKI across different PFA platforms, but comparisons among systems remain challenging because of differences in system characteristics.^3^, ^4^, ^5^, ^6^ Hemolysis is hypothesized to be influenced not only by the number of applications and inadequate catheter–tissue contact but also by variations in waveform characteristics and catheter design across different systems.^7^ This study aimed to directly compare the incidence of hemolysis, myocardial injury, and AKI among 3 single-shot PFA systems—VARIPULSE, PulseSelect, and FARAPULSE^3^, ^4^, ^5^—and assess their extent of changes compared with radiofrequency ablation (RFA) and nonablation procedures.

## Methods

### Study design

This single-center, retrospective study included 131 consecutive patients with paroxysmal or persistent atrial fibrillation (AF) who underwent first-time PFA between October 2024 and October 2025 at Nagoya University Hospital, Japan. In addition, 2 control groups were defined. 1 group comprised 23 patients who underwent RFA for AF, including redo sessions, during the same period. The other group, referred to as the nonablation group, consisted of 8 patients: 3 who underwent an electrophysiological study for syncope or palpitations without ablation and 5 who underwent coronary angiography for coronary artery evaluation. Hemolysis, myocardial, and renal function biomarkers were compared at baseline and 24 hours after the procedure in 131 patients treated with 1 of 3 PFA systems—VARIPULSE (Biosense Webster, Irvine, CA), PulseSelect (Medtronic, Minneapolis, MN), and FARAPULSE (Boston Scientific, Marlborough, MA)—and in 23 patients who underwent RFA and 8 patients who did not undergo ablation. Patients with unstable hemoglobinopathies or undergoing dialysis were excluded. The study protocol adhered to the Declaration of Helsinki and was approved by the ethics committee of Nagoya University. A written informed consent was obtained from all patients before the procedure.

### Ablation procedure

All patients received continuous oral anticoagulation therapy. Before the procedure, left atrial appendage thrombus was excluded using either contrast-enhanced computed tomography or transesophageal echocardiography. PFA procedures were conducted under deep sedation, whereas RFA procedures were performed under conscious sedation. Intravenous heparin was administered during the procedures to maintain an activated clotting time of 300–400 seconds. In all cases, 1000 mL of intravenous fluid was administered until the next day.

### PFA

PFA was initiated with a transseptal puncture using an SL0 sheath (Abbott, Plymouth, MN), which was subsequently replaced with either a Vizigo sheath (Biosense Webster), FlexCath Contour steerable sheath (Medtronic), or a FaraDrive sheath (Boston Scientific). Through these sheaths, the VARIPULSE catheter (Biosense Webster), PulseSelect catheter (Medtronic), or Farawave catheter (Boston Scientific) was introduced into the left atrium. For the VARIPULSE system, a single application was administered by the TRUPULSE generator as 1800 V bipolar biphasic square-wave pulses in 3 microsecond-scale trains, totaling approximately 250 ms. The PulseSelect system delivered 1500 V bipolar biphasic pulses using a compatible generator in 4 trains of 100–200 ms at the same site, constituting 1 application. The Farawave system delivered 1 application as 5 2000 V biphasic pulse trains (200 ms duration with 300 ms intervals) via the Farastar generator (Boston Scientific). PFA procedures were guided by the CARTO 3 system (Biosense Webster) for VARIPULSE and by the NavX system (St. Jude Medical) for both PulseSelect and FARAPULSE. Tissue contact during PFA was assessed using the EnSite™ Contact Index Module for PulseSelect and FARAPULSE and tissue proximity indication for VARIPULSE. A minimum of 12 applications per pulmonary vein was recommended for VARIPULSE, and 8 each for PulseSelect and FARAPULSE, with additional PFA applications and RFA procedures performed at the operator’s discretion.

### RFA

A transseptal puncture was performed to introduce both an SL0 sheath and an Agilis sheath (Abbott) into the left atrium. The ablation and mapping catheters were inserted through these sheaths, respectively. High-density electroanatomic mapping was conducted using either the CARTO 3 system with the Octaray catheter (Biosense Webster) or the NavX system with the HD Grid catheter (St. Jude Medical). Point-by-point lesions were created using either the QDOT MICRO (Biosense Webster) or the TactiFlex (St. Jude Medical), with an ablation power of 30–50 W applied for 10–20 seconds per lesion.

### Biochemistry analysis

Blood samples to assess markers of hemolysis, myocardial injury, and renal function from the antecubital vein on the day before ablation and 24 hours afterward. Samples were drawn without a tourniquet, using slow aspiration into collection tubes to minimize the risk of artificial hemolysis during the preanalytical phase. Biochemical and hematological tests were conducted immediately after collection. Plasma obtained from lithium heparin separation tubes was used to measure hemolysis markers (bilirubin, haptoglobin, lactate dehydrogenase [LDH]), myocardial injury markers (creatine kinase, troponin T), and renal function markers (creatinine, urea nitrogen). Haptoglobin was measured by trained staff at a designated laboratory (SRL Laboratory, Hachioji, Japan). According to the package insert, the normal range of the haptoglobin levels was 83–218 mg/dL. Significant hemolysis was defined as a ≥50% decrease in haptoglobin levels after ablation. Given the variation in the recommended number of applications required to achieve pulmonary vein isolation (PVI) among the 3 PFA systems, an application ratio was defined—calculated as the total number of applications divided by the manufacturer’s recommended number of applications (48 for VARIPULSE and 32 for PulseSelect and FARAPULSE)—to standardize biomarker changes across systems. AKI was defined and classified according to the latest Kidney Disease: Improving Global Outcomes guidelines.^8^

### Statistical analysis

Continuous variables are expressed as mean ± standard deviation or median value (interquartile ranges), and categorical variables are presented as numbers and percentages. The Student *t* test for parametric data, the Mann–Whitney *U* test for nonparametric data, and the χ^2^ test for categorical data were used to compare between the 2 groups. For comparisons involving 3 or more groups, an initial assessment of overall differences was performed using analysis of variance for normally distributed data and the Kruskal–Wallis test for nonparametric data. Significant differences were further evaluated through pairwise multiple comparisons using the Bonferroni method. Contrast data were compared using the paired *t* test or the Wilcoxon signed-rank test. The correlation between hemolytic biomarkers and the number of applications was assessed using the Pearson correlation coefficient. The coefficient of determination R^2^ was also calculated. Multiple linear regression analysis was performed to identify independent predictors of haptoglobin drop ratio in the PFA group. Receiver operating characteristic curve analysis was conducted, and the optimal cutoff point was identified using Youden’s index. All analyses were conducted using SPSS software version 30.0 (IBM Corporation). All *P* values were 2 sided, and the significance level was set at *P* < .05.

## Results

### Baseline characteristics

A total of 131 patients were treated with PFA, VARIPULSE (n = 26), PulseSelect (n = 62), and FARAPULSE PFA systems (n = 43), whereas 23 patients received RFA and 8 patients did not receive ablation. The baseline characteristics of the study population are presented in Table 1 and Supplemental Table 1. Most patients were in their late 60s, and more than half were male. The median CHADS_2_ score was 2.0 in the PulseSelect group and 1.0 in the other groups. All patients in the VARIPULSE group and 81% of those in the FARAPULSE group had paroxysmal AF, whereas 80% of those in the PulseSelect group had persistent AF. Correspondingly, B-type natriuretic peptide levels were highest in the PulseSelect group and only mildly elevated in the others. In the RFA group, approximately 60% of patients had recurrent paroxysmal AF, whereas 40% had persistent AF. The prevalence of heart failure was highest in the PulseSelect group, whereas the rates of other comorbidities were similar across all 4 groups. Mild left atrial enlargement was observed in 4 ablation groups, and left ventricular ejection fraction (LVEF) was generally preserved. Hemoglobin levels and renal function were mostly maintained, and haptoglobin levels did not differ among the 5 groups (Supplemental Table 1).

**Table 1:** Baseline characteristics of patients

Characteristics	VARIPULSE (n = 26)	PulseSelect (n = 62)	FARAPULSE (n = 43)	RFA (n = 23)	Nonablation (n = 8)	*P* value
Age, y	67.0 ± 10.3	68.2 ± 12.1	67.7 ± 10.8	66.9 ± 14.3	53.9 ± 16.2	.041
Male sex, n (%)	19 (73)	46 (74)	26 (61)	12 (52)	7 (88)	.161
Body mass index, kg/m^2^	24.3 ± 4.0	24.8 ± 3.9	23.2 ± 4.4	24.1 ± 4.0	23.4 ± 3.3	.372
CHADS_2_ score	1.0 (0.8–2.0)	2.0 (1.0–3.0)	1.0 (1.0–2.0)	1.0 (1.0–3.0)	1.0 (0–2.5)	.212
CHA_2_DS_2_-VASc score	2.0 (1.0–3.3)	3.0 (1.0–4.0)	2.0 (1.0–3.0)	2.0 (1.0–4.0)	1.0 (0–2.8)	.367
BNP levels, pg/mL	49 (15–111)	116 (60–222)	55 (30–95)	55 (22–166)	5.8 (5.8–199)	<.001
AF type, n (%)						
Paroxysmal	26 (100)	13 (21)	35 (81)	13 (57)	NA	<.001
Persistent	0 (0)	45 (73)	8 (19)	6 (26)	NA	<.001
Long-standing persistent	0 (0)	4 (6.5)	0 (0)	4 (17)	NA	.012
Comorbidity, n (%)						
Heart failure	8 (31)	39 (63)	10 (23)	9 (39)	2 (25)	<.001
Hypertension	13 (50)	38 (61)	25 (58)	12 (52)	3 (38)	.661
Diabetes mellitus	2 (7.7)	6 (9.7)	3 (7.0)	0 (0)	2 (25)	.223
Coronary artery disease	1 (3.8)	3 (4.8)	3 (7.0)	2 (8.7)	3 (38)	.061
Stroke/TIA	1 (3.8)	6 (9.7)	2 (4.7)	0 (0)	1 (13)	.378
Echocardiographic data						
Left atrial diameter, mm	39.2 ± 5.0	43.4 ± 6.3	37.3 ± 6.1	40.0 ± 6.7	35.6 ± 7.6	<.001
LVEDD, mm	49.4 ± 5.6	47.5 ± 6.0	46.0 ± 7.0	49.0 ± 7.4	46.8 ± 2.2	.207
LVEF, %	61.8 ± 6.8	58.6 ± 10.0	61.6 ± 9.1	58.9 ± 11.5	58.1 ± 11.4	.412

AF = atrial fibrillation; BNP = B-type natriuretic peptide; HF = heart failure; LVEDD = left ventricular end-diastolic diameter; LVEF = left ventricular ejection fraction; NA = not available; RFA = radiofrequency ablation; TIA = transient ischemic attack.

### Procedural characteristics

All patients in the PFA group underwent first-time PVI, whereas 83% of those in the RFA group underwent repeat ablation. Procedure duration, fluoroscopy time, additional RFA duration, and RFA energy were comparable among the 3 PFA groups. The highest rate of electrical cardioversion was observed in the PulseSelect group. The mean total number of applications differed significantly among the 3 PFA groups: 60.3 in the VARIPULSE group, 39.8 in the PulseSelect group, and 45.3 in the FARAPULSE group (Supplemental Table 2). PFA for the left atrial posterior wall was performed in 0 patients (0%) in the VARIPULSE group, 6 (9.7%) in the PulseSelect group, and 3 (7.0%) in the FARAPULSE group (*P* = .319). Cavotricuspid isthmus linear ablation using RFA was performed in 6 patients (23%) in the VARIPULSE group, 7 (11%) in the PulseSelect group, and 5 (12%) in the FARAPULSE group, also with no significant differences observed. In the RFA group, touch-up ablation for incomplete PVI was performed in 12 (52%), cavotricuspid isthmus linear ablation in 11 (48%), posterior wall isolation in 4 (17%), superior vena cava isolation in 7 (30%), and another ablation in 12 (52%). No complications were reported in any group.

### Changes in hemolysis, myocardial injury, and renal function biomarkers

**Figure 1:**
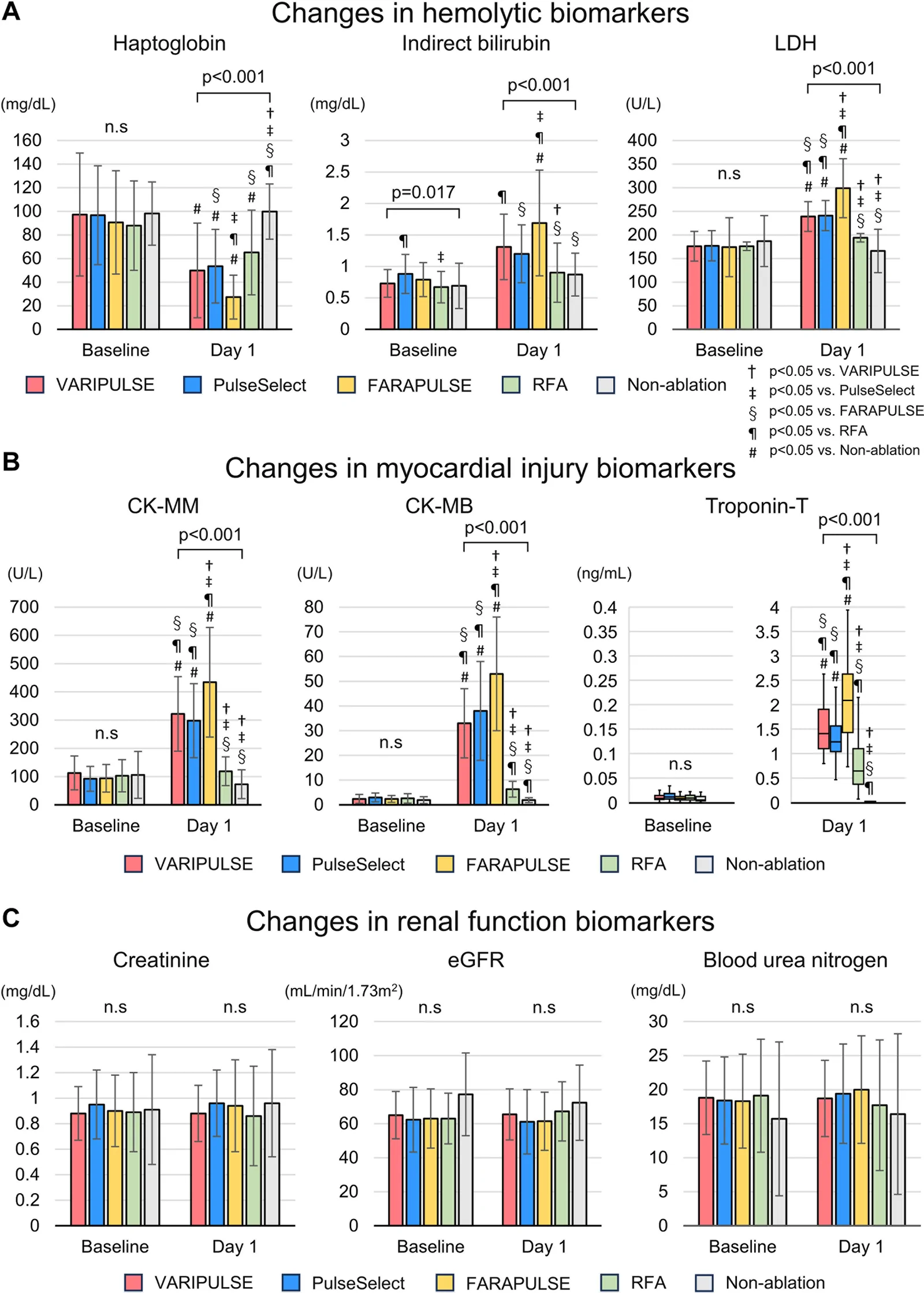
Changes in hemolytic (**A**), myocardial injury (**B**), and renal function biomarkers (**C**) among the 3 pulsed fielded ablation systems, RFA group, and nonablation group. CK-MB = creatine kinase muscle–brain type; CK-MM = creatine kinase muscle–muscle type; eGFR = estimated glomerular filtration rate; LDH = lactate dehydrogenase; RFA = radiofrequency ablation.

Haptoglobin levels significantly decreased after ablation across all 4 ablation groups, with the lowest levels observed in the FARAPULSE group and the highest in the RFA group: VARIPULSE (97.3 ± 52.1 to 49.9 ± 40.1; *P* < .001), PulseSelect (96.7 ± 41.9 to 53.5 ± 31.2; *P* < .001), FARAPULSE (90.6 ± 43.8 to 27.3 ± 18.6; *P* < .001), and RFA (87.9 ± 37.9 to 65.1 ± 35.9; *P* < .001) (Supplemental Tables 3 and 4 and Figure 1). In contrast, no significant change was observed in the nonablation group. The decrease in haptoglobin levels was lowest in the PulseSelect group and greatest in the FARAPULSE group (*P* < .001), and this pattern remained unchanged after adjustment for the application ratio. Significant hemolysis was observed in 62% of the VARIPULSE group, 48% of the PulseSelect group, 93% of the FARAPULSE group, and 13% of the RFA group, whereas no cases were observed in the nonablation group. A slight decrease in postablation hemoglobin levels was observed in both the PFA and RFA groups, whereas no such decrease was seen in the nonablation group. Levels of indirect bilirubin and LDH increased significantly after the procedure in all 4 ablation groups, with both showing the highest levels in the FARAPULSE group and the lowest in the RFA group. In the nonablation group, bilirubin levels were slightly elevated, whereas LDH levels remained within the normal range. When stratified by AF type, 3 PFA systems showed significant postablation changes in hemolytic biomarkers, with the greatest changes observed in the FARAPULSE group (Supplemental Tables 5 and 6 and Supplemental Figure 1). In the PFA group with paroxysmal AF and no additional RFA, postablation haptoglobin and LDH levels showed the greatest changes in the FARAPULSE group (Supplemental Tables 7 and 8; Supplemental Figure 2).

In all 3 PFA groups, levels of creatine kinase muscle–muscle type, creatine kinase muscle–brain type, and troponin T increased significantly after ablation. Among the 3 groups, the FARAPULSE group exhibited the highest levels of all 3 markers, even after adjustment for the application ratio. In contrast, in the RFA group, creatine kinase muscle–muscle type did not increase significantly after ablation, whereas creatine kinase muscle–brain type and troponin T increased significantly. No significant changes in myocardial injury markers were observed in the nonablation group. There was no significant increase in serum creatinine levels across the 5 groups. Similarly, no significant differences were identified among the 5 groups regarding postablation renal function markers or in the proportion of patients with elevated creatinine levels (Supplemental Table 9). Only 2 patients in the FARAPULSE group developed AKI, both classified as stage 1. In these 2 cases, baseline estimated glomerular filtration rate (eGFR) was <50 mL/min/1.73 m^2^, and both possessed multiple cardiovascular risk factors (Supplemental Table 10). Renal function in these patients returned to normal by the third day after ablation, after hydration.

### Hemolysis correlates with the number of PFA applications

**Figure 2:**
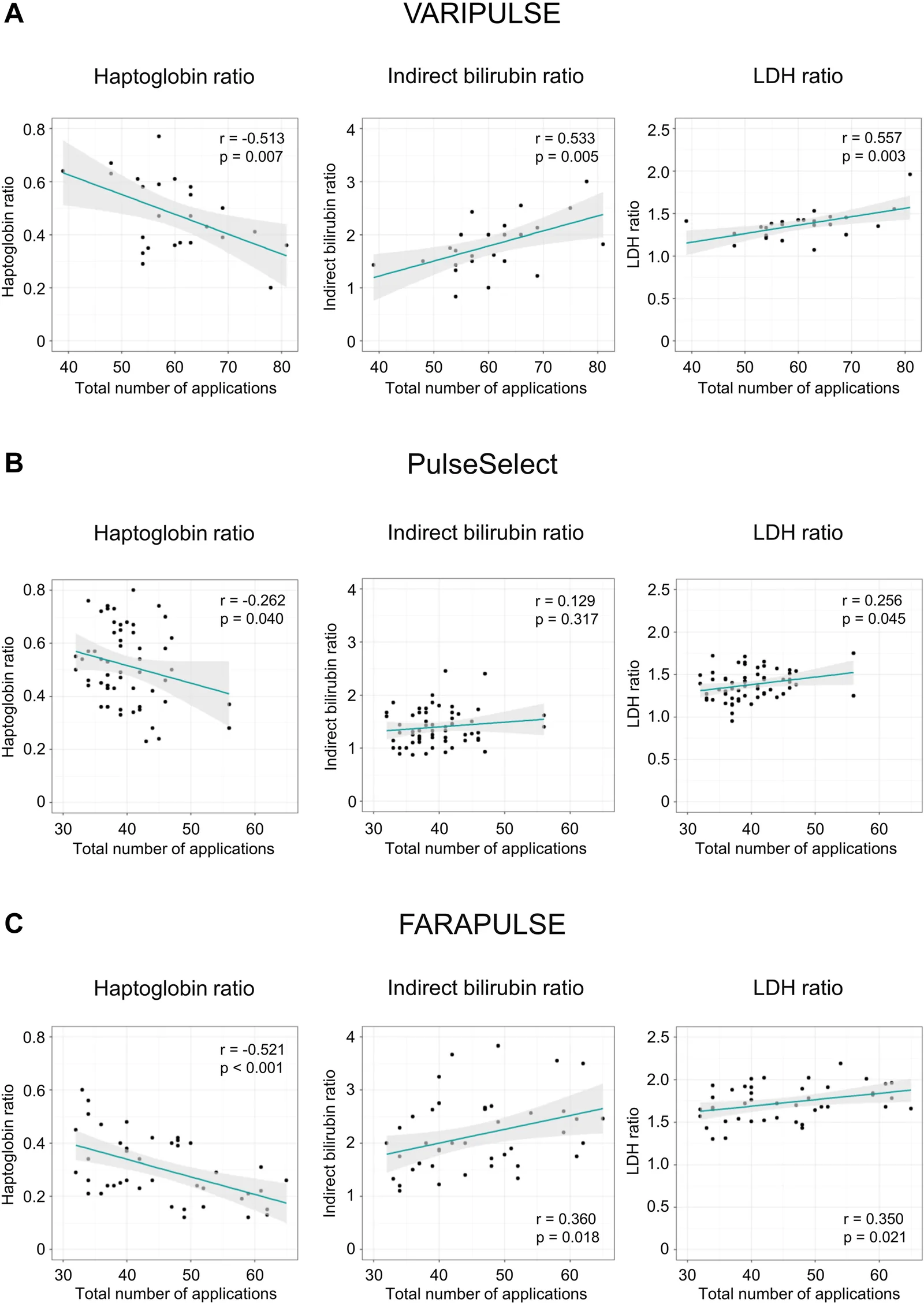
Correlation between the total number of applications and hemolytic biomarkers in the VARIPULSE (A), PulseSelect (B), and FARAPULSE (C) groups. LDH = lactate dehydrogenase.

Pearson correlation analysis was conducted to assess the association between the severity of hemolysis and the number of PFA applications. The haptoglobin ratio demonstrated a statistically significant linear correlation with the number of applications across all 3 PFA groups, showing a moderate negative correlation in the VARIPULSE and FARAPULSE groups (r = −0.51 [95% CI −0.75 to −0.16]; *P* = .007; r = −0.52 [95% CI −0.71 to −0.26]; *P* < .001, respectively) and mild negative correlation in the PulseSelect group (r = −0.26 [95% CI −0.48 to −0.01]; *P* = .040) (Figure 2). The indirect bilirubin ratio showed a moderate correlation in the VARIPULSE group (r = 0.53 [95% CI 0.18–0.76]; *P* = .005) and a mild correlation in the FARAPULSE group (r = 0.36 [95% CI 0.07–0.60]; *P* = .018), whereas no significant correlation was found in the PulseSelect group. The LDH ratio exhibited a moderate correlation in the VARIPULSE group (r = 0.56 [95% CI 0.22–0.78]; *P* = .003) and a mild correlation in the PulseSelect and FARAPULSE group (r = 0.26 [95% CI 0.01–0.48]; *P* = .045; r = 0.35 [95% CI 0.06–0.59]; *P* = .021, respectively). When stratified by AF type, LDH levels displayed a trend toward correlation in the paroxysmal AF subgroup of the PulseSelect group despite the small sample size, and in the FARAPULSE group, all hemolytic biomarkers were significantly correlated in the paroxysmal AF subgroup (Supplemental Figures 3 and 4).

Receiver operating characteristic analyses were performed to determine thresholds for the number of PFA applications associated with significant hemolysis in the PFA group with paroxysmal AF. Optimal cutoff values of 64.5, 40.5, and 35 PFA applications were identified for VARIPULSE, PulseSelect, and FARAPULSE, respectively (Supplemental Figure 5).

A multiple linear regression analysis to identify independent predictors of decreased haptoglobin ratio in the PFA groups is presented in Table 2. Variables entered into the model included age of ≥75 years, male sex, body mass index, hemoglobin level, B-type natriuretic peptide levels, eGFR of <60 mL/min/1.73 m^2^, hypertension, diabetes mellitus, left atrial diameter of >40 mm, RFA energy, and application ratio. In the final model, the application ratio (B = −0.361; 95% CI −0.476 to −0.246; *P* < .001) and body mass index (B = 0.007; 95% CI 0.001–0.014; *P* = .026) remained significant predictors. The model accounted for 25% of the variance in decreased haptoglobin ratio (R^2^ = 0.25).

**Table 2:** Multivariate regression analysis for decreased haptoglobin ratio in the PFA group

Variables	B (95% confidence interval)	β	*P* value
Application ratio	−0.361 (−0.476 to −0.246)	−0.473	<.001
Body mass index	0.007 (0.001–0.014)	0.171	.026
Intercept	0.738 (0.513–0.963)		<.001

R^2^ = 0.250. Decreased haptoglobin ratio = 0.738 − 0.361 × application ratio + 0.007 × body mass index. The decreased haptoglobin ratio was calculated as the quotient between values measured at 1 day after and before ablation.

B = unstandardized coefficients; β = standardized coefficients; eGFR = estimated glomerular filtration rate; PFA = pulsed field ablation; R = multiple correlation coefficient; RFA = radiofrequency ablation.

## Discussion

The principal findings of this study are as follows: AF ablation using 3 single-shot PFA systems resulted in significant hemolysis, whereas only mild changes were observed in the RFA group and no changes were noted in the nonablation group. The extent of hemolysis and myocardial injury was consistently greatest in the FARAPULSE group, followed by the VARIPULSE group, which exhibited a similar level as the PulseSelect group. No postablation deterioration in renal function was observed in any group, although 2 cases of AKI were reported in the FARAPULSE group. A moderate negative correlation was identified between the total number of PFA applications and the haptoglobin ratio in the VARIPULSE and FARAPULSE groups and a mild correlation in the PulseSelect group. Multivariate regression analysis identified the application ratio and body mass index as independent predictors of haptoglobin depletion.

### Hemolysis after PFA

PFA is considered safer than conventional thermal energy ablation owing to its tissue selectivity and nonthermal energy characteristics. However, the MANIFEST-17K registry reported hemolysis-induced AKI requiring dialysis in 5 patients (0.03%) of more than 17,000 treated.^1^ Acute intravascular hemolysis is characterized by a decrease in haptoglobin levels owing to a consumptive mechanism, along with increased levels of bilirubin (a by-product of heme breakdown) and LDH.^9^ We selected haptoglobin as a comparative marker because it is a reliable and well-established indicator of intravascular hemolysis.^10^ Conversely, bilirubin and LDH can be influenced by liver injury, myocardial injury, and inflammation, making haptoglobin a more robust marker of hemolysis. Variability in hemolytic susceptibility among different PFA systems is clinically important. Currently, several studies have evaluated hemolysis across different PFA systems.^3^, ^4^, ^5^^,^^11^ Our study is unique in comparing 3 single-shot PFA systems—VARIPULSE, PulseSelect, and FARAPULSE—that differ in terms of the pulse train, applied voltages, and interelectrode distance. The FARAPULSE group showed the greatest postablation decrease in haptoglobin and the largest increase in bilirubin and LDH, consistent with previous findings.^4^^,^^5^ In contrast, the PulseSelect group exhibited the smallest changes in hemolytic markers, which may be attributable to its use of the lowest voltage (1500 V). Recent research has identified that the critical threshold for achieving stable electroporation of human red blood cells with a 100-μs pulse is 1500 V/cm.^12^ The number of applications required to achieve PVI varied among the 3 PFA systems. However, even after adjustment using the application ratio, the FARAPULSE group exhibited the greatest decrease in haptoglobin. This observation may be attributable to the use of the highest voltage (2000 V) in the FARAPULSE system, and the design of basket-/flower-shaped catheters can generate an electric field at the center, which is not in direct contact with myocardial tissue, but may potentially affect circulating blood cells. Our study demonstrated that lower body mass index was independently associated with greater haptoglobin depletion, possibly reflecting smaller circulating blood volume relative to electric field exposure during PFA. Furthermore, although exploratory and restricted to patients with paroxysmal AF, the receiver operating characteristic-derived thresholds for the number of PFA applications may provide preliminary guidance regarding procedural exposure levels associated with increased hemolysis. Reportedly, PVI by RFA does not cause significant intravascular hemolysis.^2^ However, our study showed a significant decrease in haptoglobin levels after ablation in the RFA group, whereas no such change was observed in the nonablation group. These findings suggest that mild hemolysis may occur during RFA, potentially owing to hemolysis within the microvasculature of the ablated left atrial myocardium.

### Myocardial injury

PFA has been shown to significantly increase the release of myocardial injury biomarkers, and the extent of cardiac tissue damage was compared with that of RFA.^13^ Consistent with this, a previous magnetic resonance imaging study reported that acute late gadolinium enhancement after PFA was 60% greater than that observed with thermal ablation, suggesting that PFA affects not only the myocardium at the PVI line but also more surrounding, distal myocardial tissue.^14^ Given that FARAPULSE has been reported to cause greater increases in myocardial injury markers than PulseSelect,^5^ our study similarly showed that FARAPULSE resulted in the greatest myocardial damage, followed by VARIPULSE and PulseSelect, which demonstrated comparable effects. This difference is likely attributable to a combination of factors, including applied voltage, catheter design, tissue contact force, and pulse waveform.

### Renal dysfunction

Intravascular hemolysis can lead to AKI through the release of free hemoglobin into the circulation, which induces pro-oxidant effects and endothelial dysfunction. In our study, AKI was observed in 2 patients (5.6%) exclusively in the FARAPULSE group. Both had baseline eGFR values of <50 mL/min per 1.73 m^2^; 1 also had reduced LVEF, diabetes, and coronary artery disease and underwent high-dose applications, whereas the other had hypertension. Previous reports have indicated that patients with baseline renal dysfunction are more likely to develop AKI after ablation with FARAPULSE or PulseSelect.^2^^,^^11^ Hemolysis-related AKI was also reported in 7.4% of patients (4 of 54) treated with VARIPULSE, with risk factors including renal dysfunction, diabetes, hypertension, low LVEF, and high-dose applications.^3^ The present study demonstrated that patients with a low body mass index are particularly susceptible to hemolysis and that PFA-induced hemolysis increases with the number of applications in a dose-dependent manner across all PFA systems. Therefore, in addition to potential preventive strategies, such as hydration and perioperative haptoglobin administration,^15^^,^^16^ minimizing the number of applications is crucial in patients with these risk factors, regardless of the PFA system employed.

### Clinical implications

In this study, significant hemolysis was observed in 66% of patients treated with PFA, whereas AKI occurred in only 1.5%, indicating that most PFA-related hemolysis may not be associated with a high incidence of severe AKI. To mitigate this rare but serious complication, careful management is crucial, particularly in patients with renal dysfunction, high cardiovascular risk, or low body mass index. In such patients, adequate hydration and achieving durable PVI without unnecessary additional applications are essential.

### Study limitations

This study has some limitations, including the fact that most patients in the RFA control group underwent repeat ablation, whereas all patients in the PFA group underwent first-time PVI. Therefore, direct comparison between the groups and procedures may not be entirely appropriate. Second, baseline differences between device cohorts, including a higher prevalence of persistent AF and heart failure in the PulseSelect group and the exclusive inclusion of paroxysmal AF in the VARIPULSE group, may have influenced hemolytic biomarker levels; therefore, comparisons between PFA systems should be interpreted cautiously and considered hypothesis generating. However, the strong correlation between hemolytic biomarkers and the number of applications observed exclusively in the paroxysmal AF group may provide additional insight. Third, in the PulseSelect and FARAPULSE groups, additional lesion sets such as posterior wall isolation may have increased the total number of PFA applications, which could have influenced the degree of hemolysis. Fourth, catheter–tissue contact was assessed using different system-specific metrics across the 3 PFA systems, which were not directly comparable and may have influenced the degree of hemolysis. Fifth, our study did not evaluate free hemoglobin, the most sensitive marker for assessing hemolysis.^17^ Moreover, hemolysis was evaluated only using blood samples collected the day after ablation. Finally, the application ratio should be interpreted as an exploratory metric, given that differences in catheter design, pulse waveform profile, and electrode geometry among PFA systems may limit its ability to fully normalize biological exposure and could have influenced the multivariate regression analysis results regarding haptoglobin depletion. In addition, the regression model explained a modest proportion of the variance, and therefore, predictors such as body mass index should be interpreted cautiously.

## Conclusion

Intravascular hemolysis is pertinent after PFA and is related to the number of applications, application ratio, and low body mass index, with potential differences across PFA systems.

## Disclosures

Dr Yanagisawa is affiliated with a department sponsored by Medtronic Japan. The other authors have no conflicts of interest to disclose.

## Footnotes

Appendix Supplementary data associated with this article can be found in the online version at https://doi.org/10.1016/j.hroo.2026.04.009.
