# Supplementary material for: Comparative analysis of hemolysis incidence across 3 different pulsed field ablation systems
Source: Heart Rhythm O2. 2026 Apr 16;7(8):1407–15. doi: 10.1016/j.hroo.2026.04.009 (PMC13523575; doi:10.1016/j.hroo.2026.04.009)
Supplement: Supplementary file 1 [file mmc1.docx]

**Supplemental appendix**

Miyazawa *et al.*: Comparative analysis of hemolysis incidence across the three different pulsed field ablation systems

***Table of contents***

**Page 2:** *Supplemental Table 1.* Biomarker values at baseline

**Page 3:** *Supplemental Table 2.* Procedural characteristics

**Page 4:** *Supplemental Table 3*. Biomarker values on the day after ablation

**Page 5-6:** *Supplemental Table 4.* Biomarker values at baseline and the day after ablation

**Page 7:** *Supplemental Table 5.* Hemolytic biomarker levels across the PFA groups stratified according to atrial fibrillation type

**Page 8:** *Supplemental Table 6.* Hemolytic biomarker levels at baseline and the day after ablation across the PFA groups stratified by atrial fibrillation type

**Page 9:** *Supplemental Table 7.* Hemolysis biomarker levels in the PFA group with PAF and no additional RFA

**Page 10:** *Supplemental Table 8.* Hemolytic biomarker levels at baseline and the day after ablation in the PFA group with PAF and no additional RFA

**Page 11:** *Supplemental Table 9.* Clinical parameters of renal function

**Page 12:** *Supplemental Table 10.* Details of cases of acute kidney injury in the FARAPULSE group

**Page 13-14:** *Supplemental Figure 1.* Haptoglobin, indirect bilirubin, and LDH levels across the PFA groups stratified by atrial fibrillation type

**Page 15-16:** *Supplemental Figure 2.* Haptoglobin, indirect bilirubin, and LDH levels in the PFA group with PAF and no additional RFA

**Page 17-18:** *Supplemental Figure 3.* Correlation between the total number of pulse trains and hemolytic biomarkers in the PulseSelect group stratified by PAF (A) and PEF (B)

**Page 19-20:** *Supplemental Figure 4.* Correlation between the total number of pulse trains and hemolytic biomarkers in the FARAPULSE group stratified by PAF (A) and PEF (B)

**Page 21-22:** *Supplemental Figure 5.* Receiver operating characteristic analyses for PFA applications associated with significant hemolysis in the PFA group with PAF

**Supplemental Table 1.** Biomarker values at baseline

| Biomarker | VARIPULSE  (n = 26) | PulseSelect  (n = 62) | FARAPULSE  (n = 43) | RFA  (n = 23) | Non-ablation  (n = 8) | p-value |
| --- | --- | --- | --- | --- | --- | --- |
| Hemoglobin, g/dL | 13.3±1.7 | 13.5±1.7 | 13.0±1.9 | 12.9±2.2 | 14.1±2.0 | 0.409 |
| Haptoglobin, mg/dL | 97.3±52.1 | 96.7±41.9 | 90.6±43.8 | 87.9±37.9 | 98.1±26.8 | 0.876 |
| Total bilirubin, mg/dL | 0.82±0.24 | 0.98±0.33 | 0.89±0.31 | 0.74±0.28 | 0.77±0.38 | 0.017 |
| Indirect bilirubin, mg/dL | 0.73±0.22 | 0.88±0.31 | 0.79±0.27 | 0.67±0.25 | 0.69±0.36 | 0.017 |
| LDH, U/L | 176±27 | 177±26 | 174±35 | 176±33 | 187±54 | 0.977 |
| Folic acid, ng/mL | 8.2±3.3 | 7.4±2.8 | 8.0±4.7 | 7.6±2.6 | 5.9±2.4 | 0.461 |
| Iron, μg/dL | 85.9±27.8 | 101.9±44.6 | 91.8±31.7 | 79.5±35.1 | 88.3±46.1 | 0.123 |
| Potassium, mmol/L | 4.2±0.3 | 4.1±0.3 | 4.1±0.4 | 4.2±0.4 | 4.2±0.4 | 0.983 |
| Serum creatinine, mg/dL | 0.88±0.21 | 0.95±0.27 | 0.90±0.28 | 0.89±0.31 | 0.91±0.43 | 0.813 |
| eGFR, mL/min/1.73m^2^ | 65.0±13.9 | 62.3±19.0 | 63.0±17.4 | 63.0±14.9 | 77.2±24.3 | 0.253 |
| Blood urea nitrogen, mg/dL | 18.8±5.4 | 18.4±6.4 | 18.3±6.9 | 19.1±8.3 | 15.7±11.3 | 0.830 |
| CK-MM, U/L | 113±60 | 92±44 | 94±49 | 103±57 | 106±83 | 0.473 |
| CK-MB, U/L | 2.4±1.8 | 3.0±1.8 | 2.4±1.4 | 2.6±1.9 | 1.9±1.4 | 0.208 |
| Troponin T, ng/mL | 0.008 (0.007, 0.014) | 0.011 (0.007, 0.019) | 0.008 (0.006, 0.012) | 0.010 (0.006, 0.015) | 0.005 (0.004, 0.014) | 0.194 |
| C-reactive protein, mg/dL | 0.035 (0.030, 0.100) | 0.075 (0.040, 0.153) | 0.050 (0.030, 0.210) | 0.050 (0.020, 0.160) | 0.030 (0.023, 0.080) | 0.130 |

CK-MB, creatine kinase muscle-brain type; CK-MM, creatine kinase muscle-muscle type; eGFR, estimated glomerular filtration rate; LDH, lactate dehydrogenase; RFA, radiofrequency ablation.

**Supplemental Table 2.** Procedural characteristics

|  | VARIPULSE  (n=26) | PulseSelect  (n=62) | FARAPULSE  (n=43) | RFA  (n=23) | Non-ablation  (n=8) | p-value |
| --- | --- | --- | --- | --- | --- | --- |
| Index ablation | 26 (100) | 62 (100) | 43 (100) | 4 (17) | NA | <0.001 |
| Procedure duration, min | 74.5±20.1 | 70.8±19.5 | 66.5±19.8 | 116.7±24.1 | 36.1±20.5 | <0.001 |
| Fluoroscopy duration, min | 20.6±6.8 | 23.7±10.3 | 22.5±8.1 | 28.9±14.5 | 6.8±1.9 | 0.076 |
| RFA duration, min | 4.4 (3.0, 10.0) | 4.9 (3.0, 10.5) | 6.3 (4.5, 9.9) | 18.4 (13.0, 36.0) | NA | *0.765 |
| RFA energy, J | 8283 (6280, 19085) | 11574 (7600, 20156) | 13412 (9402, 21388) | 47785 (20963, 83005) | NA | *0.706 |
| Electrical cardioversion | 4 (15) | 35 (57) | 4 (9.3) | 5 (22) | NA | <0.001 |
| Total number of pulse trains | 180±28 | 160±20 | 226±48 | NA | NA | *<0.001 |
| Total number of applications | 60.3±9.5 | 39.8±5.0 | 45.3±9.7 | NA | NA | *<0.001 |
| Application ratio | 1.26±0.04 | 1.25±0.02 | 1.41±0.30 | NA | NA | *<0.006 |
| Sinus rhythm at discharge | 25 (96) | 55 (89) | 43 (100) | 18 (78) | NA | *0.046 |
| Posterior wall isolation with PFA | 0 (0) | 6 (9.7) | 3 (7.0) | NA | NA | *0.319 |
| Additional RFA |  |  |  |  |  |  |
| Touch-up for incomplete PVI | 0 (0) | 0 (0) | 0 (0) | 12 (52) | NA | <0.001 |
| CTI line | 6 (23) | 7 (11) | 5 (12) | 11 (48) | NA | 0.002 |
| Posterior wall isolation | 0 (0) | 0 (0) | 0 (0) | 4 (17) | NA | <0.001 |
| SVC isolation | 0 (0) | 0 (0) | 0 (0) | 7 (30) | NA | <0.001 |
| Other | 2 (7.7) | 2 (3.2) | 0 (0) | 12 (52) | NA | <0.001 |
| Complication | 0 (0) | 0 (0) | 0 (0) | 0 (0) | 0 (0) | NA |

*p-values indicate comparisons among three groups: Varipulse, PulseSelect, and Farapulse. Others reflect comparisons among four groups: Varipulse, PulseSelect, Farapulse, and RFA. The application ratio was defined as the ratio of the total number of applications to the recommended number of applications (48 for VARIPULSE and 32 for PulseSelect and FARAPULSE). Other RFA procedures in the RFA group included roof line ablation (n=2), mitral isthmus ablation (n=3), anterior wall ablation (n=3), non-PV foci ablation (n=7), left atrial ablation for incidentally detected atrial tachycardia (n=2), and slow pathway ablation for incidentally detected AVNRT (n=1). In the PFA group, other additional RFA ablations included mitral isthmus ablation (n=1), anterior wall ablation (n=1), right atrial ablation for incidentally detected atrial tachycardia (n=1), and slow pathway ablation for incidentally detected AVNRT (n=1).

AVNRT, atrioventricular nodal reentrant tachycardia; CTI, cavo-tricuspid isthmus; PFA, pulsed field ablation; PVI, pulmonary vein isolation; RFA, radiofrequency ablation; SVC, superior vena cava.

**Supplemental Table 3.** Biomarker values on the day after ablation

| Biomarker | VARIPULSE  (n=26) | PulseSelect  (n=62) | FARAPULSE  (n=43) | RFA  (n=23) | Non-ablation  (n=8) | p-value |
| --- | --- | --- | --- | --- | --- | --- |
| Hemoglobin, g/dL | 12.6±1.7 | 12.6±1.6 | 12.3±1.7 | 12.1±2.2 | 14.4±1.9 | 0.028 |
| Significant hemolysis* | 16 (62) | 30 (48) | 40 (93) | 3 (13) | 0 (0) | <0.001 |
| Haptoglobin, mg/dL | 49.9±40.1 | 53.5±31.2 | 27.3±18.6 | 65.1±35.9 | 99.8±23.4 | <0.001 |
| ΔHaptoglobin, mg/dL | -47.4±21.5 | -43.2±21.7 | -63.3±33.1 | -22.7±14.2 | 1.6±7.5 | <0.001 |
| ΔHaptoglobin per application, mg/dL | -0.79±0.33 | -1.09±0.54 | -1.45±0.78 | NA | NA | <0.001 |
| ΔHaptoglobin per application ratio, mg/dL | -38.1±15.9 | -35.0±17.4 | -46.4±24.9 | NA | NA | 0.040 |
| Total bilirubin, mg/dL | 1.42±0.54 | 1.32±0.48 | 1.89±0.93 | 1.03±0.46 | 0.96±0.33 | <0.001 |
| Indirect bilirubin, mg/dL | 1.31±0.52 | 1.20±0.46 | 1.69±0.84 | 0.90±0.47 | 0.87±0.34 | <0.001 |
| LDH, U/L | 239±33 | 241±37 | 299±63 | 194±38 | 166±46 | <0.001 |
| Folic acid, ng/mL | 7.1±3.2 | 6.5±2.5 | 7.1±3.0 | 7.1±2.8 | 5.8±1.9 | 0.634 |
| Iron, μg/dL | 76.1±37.5 | 76.8±50.6 | 128.4±56.8 | 61.7±29.7 | 121.5±54.9 | <0.001 |
| Potassium, mmol/L | 4.1±0.3 | 4.1±0.4 | 4.0±0.4 | 4.0±0.4 | 4.3±0.4 | 0.253 |
| Serum creatinine, mg/dL | 0.88±0.22 | 0.96±0.26 | 0.94±0.36 | 0.86±0.39 | 0.96±0.42 | 0.700 |
| eGFR, mL/min/1.73m^2^ | 65.4±15.0 | 61.1±18.9 | 61.4±17.1 | 67.2±17.4 | 72.3±22.1 | 0.303 |
| Blood urea nitrogen, mg/dL | 18.7±5.6 | 19.4±7.3 | 20.0±7.9 | 17.7±9.6 | 16.4±11.8 | 0.675 |
| CK-MM, U/L | 322±132 | 298±131 | 434±194 | 119±51 | 73±51 | <0.001 |
| CK-MB, U/L | 33±14 | 38±20 | 53±23 | 6.3±3.2 | 1.9±1.0 | <0.001 |
| ΔCK-MB per application ratio, U/L | 26±14 | 28±16 | 37±18 | NA | NA | 0.006 |
| Troponin T, ng/mL | 1.41 (1.10, 1.91) | 1.24 (1.04, 1.57) | 2.09 (1.43, 2.63) | 0.64 (0.37, 1.10) | 0.016 (0.007, 0.021) | <0.001 |
| ΔTroponin T per application ratio, ng/mL | 1.11 (0.87, 1.64) | 1.03 (0.80, 1.21) | 1.40 (1.14, 1.86) | NA | NA | <0.001 |
| C-reactive protein, mg/dL | 0.63 (0.35, 0.82) | 0.57 (0.40, 1.05) | 0.84 (0.49, 1.29) | 0.54 (0.22, 0.95) | 0.07 (0.04, 0.13) | <0.001 |

*Significant hemolysis was defined as a ≥50% decrease in haptoglobin levels after ablation. CK-MB, creatine kinase muscle-brain type; CK-MM, creatine kinase muscle-muscle type; eGFR, estimated glomerular filtration rate; LDH, lactate dehydrogenase; RFA, radiofrequency ablation.

**Supplemental Table 4.** Biomarker values at baseline and the day after ablation

| Biomarker | Baseline | Day after ablation | p-value |
| --- | --- | --- | --- |
| VARIPULSE (n = 26) |  |  |  |
| Hemoglobin, g/dL | 13.3±1.7 | 12.6±1.7 | <0.001 |
| Haptoglobin, mg/dL | 97.3±52.1 | 49.9±40.1 | <0.001 |
| Total bilirubin, mg/dL | 0.82±0.24 | 1.42±0.54 | <0.001 |
| Indirect bilirubin, mg/dL | 0.73±0.22 | 1.31±0.52 | <0.001 |
| LDH, U/L | 176±27 | 239±33 | <0.001 |
| Folic acid, ng/mL | 8.2±3.3 | 7.1±3.2 | <0.001 |
| Iron, μg/dL | 85.9±27.8 | 76.1±37.5 | 0.202 |
| Potassium, mmol/L | 4.2±0.3 | 4.1±0.3 | 0.230 |
| Serum creatinine, mg/dL | 0.88±0.21 | 0.88±0.22 | 0.918 |
| eGFR, mL/min/1.73m^2^ | 65.0±13.9 | 65.4±15.0 | 0.817 |
| Blood urea nitrogen, mg/dL | 18.8±5.4 | 18.7±5.6 | 0.911 |
| CK-MM, U/L | 113±60 | 322±132 | <0.001 |
| CK-MB, U/L | 2.4±1.8 | 33±14 | <0.001 |
| Troponin T, ng/mL | 0.008 (0.007, 0.014) | 1.41 (1.10, 1.91) | <0.001 |
| C-reactive protein, mg/dL | 0.035 (0.030, 0.100) | 0.63 (0.35, 0.82) | <0.001 |
|  |  |  |  |
| PulseSelect (n = 62) |  |  |  |
| Hemoglobin, g/dL | 13.5±1.7 | 12.6±1.6 | <0.001 |
| Haptoglobin, mg/dL | 96.7±41.9 | 53.5±31.2 | <0.001 |
| Total bilirubin, mg/dL | 0.98±0.33 | 1.32±0.48 | <0.001 |
| Indirect bilirubin, mg/dL | 0.88±0.31 | 1.20±0.46 | <0.001 |
| LDH, U/L | 177±26 | 241±37 | <0.001 |
| Folic acid, ng/mL | 7.4±2.8 | 6.5±2.5 | <0.001 |
| Iron, μg/dL | 101.9±44.6 | 76.8±50.6 | 0.005 |
| Potassium, mmol/L | 4.1±0.3 | 4.1±0.4 | 1.000 |
| Serum creatinine, mg/dL | 0.95±0.27 | 0.96±0.26 | 0.452 |
| eGFR, mL/min/1.73m^2^ | 62.3±19.0 | 61.1±18.9 | 0.292 |
| Blood urea nitrogen, mg/dL | 18.4±6.4 | 19.4±7.3 | 0.094 |
| CK-MM, U/L | 92±44 | 298±131 | <0.001 |
| CK-MB, U/L | 3.0±1.8 | 38±20 | <0.001 |
| Troponin T, ng/mL | 0.011 (0.007, 0.019) | 1.24 (1.04, 1.57) | <0.001 |
| C-reactive protein, mg/dL | 0.075 (0.040, 0.153) | 0.57 (0.40, 1.05) | <0.001 |
|  |  |  |  |
| FARAPULSE (n = 43) |  |  |  |
| Hemoglobin, g/dL | 13.0±1.9 | 12.3±1.7 | <0.001 |
| Haptoglobin, mg/dL | 90.6±43.8 | 27.3±18.6 | <0.001 |
| Total bilirubin, mg/dL | 0.89±0.31 | 1.89±0.93 | <0.001 |
| Indirect bilirubin, mg/dL | 0.79±0.27 | 1.69±0.84 | <0.001 |
| LDH, U/L | 174±35 | 299±63 | <0.001 |
| Folic acid, ng/mL | 8.0±4.7 | 7.1±3.0 | 0.074 |
| Iron, μg/dL | 91.8±31.7 | 128.4±56.8 | 0.001 |
| Potassium, mmol/L | 4.1±0.4 | 4.0±0.4 | 0.029 |
| Serum creatinine, mg/dL | 0.90±0.28 | 0.94±0.36 | 0.100 |
| eGFR, mL/min/1.73m^2^ | 63.0±17.4 | 61.4±17.1 | 0.095 |
| Blood urea nitrogen, mg/dL | 18.3±6.9 | 20.0±7.9 | 0.016 |
| CK-MM, U/L | 94±49 | 434±194 | <0.001 |
| CK-MB, U/L | 2.4±1.4 | 53±23 | <0.001 |
| Troponin T, ng/mL | 0.008 (0.006, 0.012) | 2.09 (1.43, 2.63) | <0.001 |
| C-reactive protein, mg/dL | 0.050 (0.030, 0.210) | 0.84 (0.49, 1.29) | <0.001 |
|  |  |  |  |
| RFA (n = 23) |  |  |  |
| Hemoglobin, g/dL | 12.9±2.2 | 12.1±2.2 | <0.001 |
| Haptoglobin, mg/dL | 87.9±37.9 | 65.1±35.9 | <0.001 |
| Total bilirubin, mg/dL | 0.74±0.28 | 1.03±0.46 | <0.001 |
| Indirect bilirubin, mg/dL | 0.67±0.25 | 0.90±0.47 | 0.002 |
| LDH, U/L | 176±33 | 194±38 | 0.002 |
| Folic acid, ng/mL | 7.6±2.6 | 7.1±2.8 | 0.059 |
| Iron, μg/dL | 79.5±35.1 | 61.7±29.7 | 0.033 |
| Potassium, mmol/L | 4.2±0.4 | 4.0±0.4 | 0.001 |
| Serum creatinine, mg/dL | 0.89±0.31 | 0.86±0.39 | 0.339 |
| eGFR, mL/min/1.73m^2^ | 63.0±14.9 | 67.2±17.4 | 0.010 |
| Blood urea nitrogen, mg/dL | 19.1±8.3 | 17.7±9.6 | 0.191 |
| CK-MM, U/L | 103±57 | 119±51 | 0.076 |
| CK-MB, U/L | 2.6±1.9 | 6.3±3.2 | <0.001 |
| Troponin T, ng/mL | 0.010 (0.006, 0.015) | 0.64 (0.37, 1.10) | <0.001 |
| C-reactive protein, mg/dL | 0.050 (0.020, 0.160) | 0.54 (0.22, 0.95) | <0.001 |
|  |  |  |  |
| Non-ablation (n = 8) |  |  |  |
| Hemoglobin, g/dL | 14.1±2.0 | 14.4±1.9 | 0.403 |
| Haptoglobin, mg/dL | 98.1±26.8 | 99.8±23.4 | 0.561 |
| Total bilirubin, mg/dL | 0.77±0.38 | 0.96±0.33 | 0.004 |
| Indirect bilirubin, mg/dL | 0.69±0.36 | 0.87±0.34 | 0.011 |
| LDH, U/L | 187±54 | 166±46 | 0.036 |
| Folic acid, ng/mL | 5.9±2.4 | 5.8±1.9 | 0.707 |
| Iron, μg/dL | 88.3±46.1 | 121.5±54.9 | 0.199 |
| Potassium, mmol/L | 4.2±0.4 | 4.3±0.4 | 0.155 |
| Serum creatinine, mg/dL | 0.91±0.43 | 0.96±0.42 | 0.067 |
| eGFR, mL/min/1.73m^2^ | 77.2±24.3 | 72.3±22.1 | 0.088 |
| Blood urea nitrogen, mg/dL | 15.7±11.3 | 16.4±11.8 | 0.519 |
| CK-MM, U/L | 106±83 | 73±51 | 0.038 |
| CK-MB, U/L | 1.9±1.4 | 1.9±1.0 | 1.000 |
| Troponin T, ng/mL | 0.005 (0.004, 0.014) | 0.016 (0.007, 0.021) | 0.012 |
| C-reactive protein, mg/dL | 0.030 (0.023, 0.080) | 0.07 (0.04, 0.13) | 0.041 |

CK-MB, creatine kinase muscle-brain type; CK-MM, creatine kinase muscle-muscle type; eGFR, estimated glomerular filtration rate; LDH, lactate dehydrogenase; RFA, radiofrequency ablation.

**Supplemental Table 5.** Hemolytic biomarker levels across the PFA groups stratified according to arial fibrillation type

| Biomarker | VARIPULSE, PAF (n=26) | PulseSelect, PAF (n=13) | PulseSelect, PEF (n=49) | FARAPULSE, PAF (n=35) | FARAPULSE, PEF (n=8) | p-value |
| --- | --- | --- | --- | --- | --- | --- |
| Baseline | | | | | | |
| Haptoglobin, mg/dL | 97.3±52.1 | 99.7±45.2 | 95.9±41.4 | 87.7±47.5 | 103.3±17.8 | 0.837 |
| Total bilirubin, mg/dL | 0.82±0.24 | 0.90±0.31 | 0.99±0.34 | 0.88±0.32 | 0.93±0.25 | 0.173 |
| Indirect bilirubin, mg/dL | 0.73±0.22 | 0.82±0.28 | 0.90±0.31 | 0.78±0.28 | 0.85±0.21 | 0.136 |
| LDH, U/L | 176±27 | 177±21 | 177±28 | 172±37 | 183±28 | 0.905 |
|  | | | | | | |
| 1 day post-ablation | | | | | | |
| Haptoglobin, mg/dL | 49.9±40.1 | 57.0±27.7 | 52.6±32.3 | 26.1±19.8 | 32.6±11.4 | <0.001 |
| ΔHaptoglobin, mg/dL | -47.4±21.5 | -42.7±21.9 | -43.4±21.8 | -61.6±36.0 | -70.6±14.1 | 0.001 |
| ΔHaptoglobin per application, mg/dL | -0.79±0.33 | -1.07±0.60 | -1.10±0.54 | -1.44±0.84 | -1.49±0.47 | <0.001 |
| ΔHaptoglobin per application ratio, mg/dL | -38.1±15.9 | -34.3±19.0 | -35.2±17.2 | -46.1±26.8 | -47.7±14.9 | 0.119 |
| Total bilirubin, mg/dL | 1.42±0.54 | 1.15±0.42 | 1.36±0.48 | 1.86±0.97 | 2.03±0.77 | 0.007 |
| Indirect bilirubin, mg/dL | 1.31±0.52 | 1.05±0.39 | 1.24±0.47 | 1.65±0.87 | 1.89±0.69 | 0.008 |
| LDH, U/L | 239±33 | 255±33 | 238±37 | 300±64 | 296±63 | <0.001 |

LDH, lactate dehydrogenase; PAF, paroxysmal atrial fibrillation; PEF, persistent atrial fibrillation; PFA, pulsed field ablation.

**Supplemental Table 6.** Hemolytic biomarker levels at baseline and the day after ablation across PFA groups stratified according to atrial fibrillation type

| Biomarker | Baseline | Day after ablation | p-value |
| --- | --- | --- | --- |
| VARIPULSE, PAF (n = 26) |  |  |  |
| Haptoglobin, mg/dL | 97.3±52.1 | 49.9±40.1 | <0.001 |
| Total bilirubin, mg/dL | 0.82±0.24 | 1.42±0.54 | <0.001 |
| Indirect bilirubin, mg/dL | 0.73±0.22 | 1.31±0.52 | <0.001 |
| LDH, U/L | 176±27 | 239±33 | <0.001 |
|  |  |  |  |
| PulseSelect, PAF (n = 13) |  |  |  |
| Haptoglobin, mg/dL | 99.7±45.2 | 57.0±27.7 | <0.001 |
| Total bilirubin, mg/dL | 0.90±0.31 | 1.15±0.42 | 0.005 |
| Indirect bilirubin, mg/dL | 0.82±0.28 | 1.05±0.39 | 0.005 |
| LDH, U/L | 177±21 | 255±33 | <0.001 |
|  |  |  |  |
| PulseSelect, PEF (n = 45) |  |  |  |
| Haptoglobin, mg/dL | 95.9±41.4 | 52.6±32.3 | <0.001 |
| Total bilirubin, mg/dL | 0.99±0.34 | 1.36±0.48 | <0.001 |
| Indirect bilirubin, mg/dL | 0.90±0.31 | 1.24±0.47 | <0.001 |
| LDH, U/L | 177±28 | 238±37 | <0.001 |
|  |  |  |  |
| FARAPULSE, PAF (n = 35) |  |  |  |
| Haptoglobin, mg/dL | 87.7±47.5 | 26.1±19.8 | <0.001 |
| Total bilirubin, mg/dL | 0.88±0.32 | 1.86±0.97 | <0.001 |
| Indirect bilirubin, mg/dL | 0.78±0.28 | 1.65±0.87 | <0.001 |
| LDH, U/L | 172±37 | 300±64 | <0.001 |
|  |  |  |  |
| FARAPULSE, PEF (n = 8) |  |  |  |
| Haptoglobin, mg/dL | 103.3±17.8 | 32.6±11.4 | <0.001 |
| Total bilirubin, mg/dL | 0.93±0.25 | 2.03±0.77 | <0.001 |
| Indirect bilirubin, mg/dL | 0.85±0.21 | 1.89±0.69 | <0.001 |
| LDH, U/L | 183±28 | 296±63 | <0.001 |

LDH, lactate dehydrogenase; PAF, paroxysmal atrial fibrillation; PEF, persistent atrial fibrillation; PFA, pulsed field ablation.

**Supplemental Table 7.** Hemolysis biomarker levels in the PFA group with PAF and no additional RFA

| Biomarker | VARIPULSE  (n=19) | PulseSelect  (n=11) | FARAPULSE  (n=30) | p-value |
| --- | --- | --- | --- | --- |
| Baseline |  |  |  |  |
| Haptoglobin, mg/dL | 90.9±49.6 | 93.0±46.0 | 86.3±49.0 | 0.907 |
| Total bilirubin, mg/dL | 0.86±0.24 | 0.95±0.27 | 0.84±0.33 | 0.612 |
| Indirect bilirubin, mg/dL | 0.77±0.22 | 0.86±0.26 | 0.75±0.28 | 0.523 |
| LDH, U/L | 170±26 | 172±19 | 168±27 | 0.896 |
|  |  |  |  |  |
| 1 day post-ablation | ± | ± | ± |  |
| Haptoglobin, mg/dL | 45.6±35.1 | 52.9±28.0 | 24.4±19.3 | 0.006 |
| ΔHaptoglobin, mg/dL | -45.3±18.4 | -40.1±23.0 | -61.9±37.5 | 0.062 |
| ΔHaptoglobin per application, mg/dL | -0.77±0.34 | -0.97±0.59 | -1.42±0.85 | 0.004 |
| ΔHaptoglobin per application ratio, mg/dL | -37.2±16.2 | -31.1±18.9 | -45.5±27.2 | 0.166 |
| Total bilirubin, mg/dL | 1.53±0.54 | 1.19±0.40 | 1.80±0.96 | 0.020 |
| Indirect bilirubin, mg/dL | 1.42±0.52 | 1.08±0.38 | 1.58±0.83 | 0.123 |
| LDH, U/L | 231±32 | 249±32 | 294±50 | <0.001 |

LDH, lactate dehydrogenase; PAF, paroxysmal atrial fibrillation; PFA, pulsed field ablation; RFA, radiofrequency ablation.

**Supplemental Table 8.** Hemolytic biomarker levels at baseline and the day after ablation in the PFA group with PAF and no additional RFA

| Biomarker | Baseline | Day after ablation | p value |
| --- | --- | --- | --- |
| VARIPULSE (n = 19) |  |  |  |
| Haptoglobin, mg/dL | 90.9±49.6 | 45.6±35.1 | <0.001 |
| Total bilirubin, mg/dL | 0.86±0.24 | 1.53±0.54 | <0.001 |
| Indirect bilirubin, mg/dL | 0.77±0.22 | 1.42±0.52 | <0.001 |
| LDH, U/L | 170±26 | 231±32 | <0.001 |
|  |  |  |  |
| PulseSelect (n = 11) |  |  |  |
| Haptoglobin, mg/dL | 93.0±46.0 | 52.9±28.0 | <0.001 |
| Total bilirubin, mg/dL | 0.95±0.27 | 1.19±0.40 | 0.017 |
| Indirect bilirubin, mg/dL | 0.86±0.26 | 1.08±0.38 | 0.017 |
| LDH, U/L | 172±19 | 249±32 | <0.001 |
|  |  |  |  |
| FARAPULSE (n = 30) |  |  |  |
| Haptoglobin, mg/dL | 86.3±49.0 | 24.4±19.3 | <0.001 |
| Total bilirubin, mg/dL | 0.84±0.33 | 1.80±0.96 | <0.001 |
| Indirect bilirubin, mg/dL | 0.75±0.28 | 1.58±0.83 | <0.001 |
| LDH, U/L | 168±27 | 294±50 | <0.001 |

LDH, lactate dehydrogenase; PAF, paroxysmal atrial fibrillation; PFA, pulsed field ablation; RFA, radiofrequency ablation.

**Supplemental Table 9.** Clinical parameters of renal function

|  | VARIPULSE  (n=26) | PulseSelect  (n=62) | FARAPULSE  (n=43) | RFA  (n=23) | Non-ablation  (n=8) | p-value |
| --- | --- | --- | --- | --- | --- | --- |
| Creatinine ratio (Day1/baseline) | 1.00±0.10 | 1.02±0.12 | 1.03±0.11 | 0.96±0.10 | 1.06±0.08 | 0.060 |
| ΔCreatinine, mg/dL | 0 (-0.06, 0.08) | 0.04 (-0.06, 0.09) | 0.02 (-0.03, 0.08) | -0.02 (-0.01, 0.03) | 0.03 (0, 0.10) | 0.173 |
| ΔCreatinine >0 | 13 (50) | 35 (57) | 25 (58) | 10 (44) | 6 (75) | 0.578 |
| Acute kidney injury | 0(0) | 0(0) | 2 (5.6) | 0 (0) | 0 (0) | 0.256 |
| Stage 1 | 0(0) | 0(0) | 2 (5.6) | 0 (0) | 0 (0) | 0.256 |
| Stage 2 | 0(0) | 0(0) | 0(0) | 0 (0) | 0 (0) | NA |
| Stage 3 | 0(0) | 0(0) | 0(0) | 0 (0) | 0 (0) | NA |

RFA, radiofrequency ablation.

**Supplemental Table 10.** Details regarding cases of acute kidney injury in the FARAPULSE group

| Case | Age, years | Sex | BMI,  kg/m^2^ | LVEF,  % | Total number of applications,  n | Application ratio | Cr  baseline-d1-d3,  mg/dL | eGFR  baseline-d1-d3,  mL/min/1.73m^2^ | Haptoglobin  baseline-d1,  mg/dL | Indirect bilirubin baseline-d1,  mg/dL |
| --- | --- | --- | --- | --- | --- | --- | --- | --- | --- | --- |
| 1 | 72 | Male | 23.3 | 20.4 | 52 | 1.63 | 1.99-2.71-1.86 | 26.8-19.1-28.8 | 172-28 | 0.7-1.10 |
| 2 | 76 | Male | 26.0 | 64.0 | 40 | 1.25 | 1.11-1.44-1.06 | 49.9-37.6-52.5 | 67-25 | 1.20-3.90 |

| Case | LDH baseline-d1,  U/L | CKD (eGFR<60) | HF | HT | Diabetes | CAD | Stroke/TIA | CHADS_2_ score | CHA_2_DS_2_-VASc score |
| --- | --- | --- | --- | --- | --- | --- | --- | --- | --- |
| 1 | 204-390 | (+) | (+) | (-) | (+) | (+) | (-) | 2 | 4 |
| 2 | 153-307 | (+) | (-) | (+) | (-) | (-) | (-) | 2 | 3 |

BMI, body mass index; CAD, coronary artery disease; CKD, chronic kidney disease; Cr, creatinine; d, day; eGFR, estimated glomerular filtration rate; HF, heart failure; HT, hypertension; LDH, lactate dehydrogenase; LVEF, left ventricular ejection fraction; TIA, transient ischemic attack.

**Supplemental Figure 1**

***Supplemental Figure 1***

**Haptoglobin, indirect bilirubin, and LDH levels across the PFA groups stratified by atrial fibrillation type**

LDH, lactate dehydrogenase; PAF, paroxysmal atrial fibrillation; PEF, persistent atrial fibrillation; PFA, pulsed field ablation.

**Supplemental Figure 2**

***Supplemental Figure 2***

**Haptoglobin, indirect bilirubin, and LDH levels in the PFA group with PAF and no additional RFA**

LDH, lactate dehydrogenase; PAF, paroxysmal atrial fibrillation; PFA, pulsed field ablation; RFA, radiofrequency ablation.

**Supplemental Figure 3**

***Supplemental Figure 3***

**Correlation between the total number of applications and hemolytic biomarkers in the PulseSelect group stratified by PAF (A) and PEF (B)**

LDH, lactate dehydrogenase; PAF, paroxysmal atrial fibrillation; PEF, persistent atrial fibrillation.

**Supplemental Figure 4**

***Supplemental Figure 4***

**Correlation between the total number of applications and hemolytic biomarkers in the FARAPULSE group stratified by PAF (A) and PEF (B)**

LDH, lactate dehydrogenase; PAF, paroxysmal atrial fibrillation; PEF, persistent atrial fibrillation.

**Supplemental Figure 5**

***Supplemental Figure 5***

**Receiver operating characteristic analyses for PFA applications associated with the significant hemolysis in the PFA group with PAF**

AUC, area under the curve; CI, confidence interval; PAF, paroxysmal atrial fibrillation; PFA, pulsed field ablation.
